# Lectures on the Exploration and Treatment of Diseases of the Chest

**Published:** 1840-05-23

**Authors:** W. W. Gerhard


### MEDICAL EXAMINER.

DEVOTED TO MEDICINE, SURGERY, AND THE COLLATERAL SCIENCES.

No. 21.] PHILADELPHIA, SATURDAY, MAY 23, 1840. [Vol. III.

### LECTURES ON THE EXPLORATION AND TREATMENT OF DISEASES OF THE CHEST.

BY W. W. GERHARD, M. D.

Lecture II.—*Conformation of Chest—Circumstances attending it—Mode of Examination—Mensuration—Succussion.*

My first lecture was designed to prevent a frequent source of error, which often produces either an obvious or a concealed influence upon the mind. This is a desire to lay too much stress upon a single set of symptoms, to the neglect of others, and to examine a disease of a part of the body as if it were nearly or altogether unconnected with the same or with different disorders which attack other organs and tissues. In commencing, therefore, a course of lectures which are founded upon the positive evidence of anatomical lesions, and of the corresponding physical signs, I would put you upon your guard against too anatomical a view of the subject, too exclusive a study of lesions, and warn you against allowing the results of disease to be confounded with disease itself, or the physical signs which constitute the key to so many important researches, from being mistaken for actual diagnosis. It is the deductions from the whole of the physical signs and functional symptoms which constitutes the diagnosis, not the naked examination of a single set of them. This may seem a matter which is too trivial to attract much notice; but in practice it is of much moment, and the errors which I have seen from a neglect of it are frequent, and very readily committed. It may seem to you that this is reasoning against myself, as it were, and attacking the subject upon which I lay so much stress; but, in professing to give a course of lectures on diagnosis and treatment, I am necessarily led to an enlarged study of pathology, and to the view of the subject which seems to me most consistent with facts,—that is, one embracing the relation of the phenomena one to another. I am also unwilling that you should diminish the value of positive observation, by drawing any inferences which the actual state of the subject will not fully warrant; this would be the case, if, at the commencement of your studies, you fall into a contracted, imperfect mode of reasoning.

After warning you thus earnestly against the abuse of the physical signs, I may proceed to point out the best method of avoiding or overcoming the difficulties which you meet with at the commencement of your studies. They depend in a great degree upon the want of fixed starting points from which the study of the subject may begin. These points are readily enough learned by those who possess the advantages of examining for themselves, after the sounds have been pointed out by their teacher; and they are, perhaps, less necessary for you. This is not the case with many of those who may read, but do not hear these lectures; and it is therefore necessary to explain fully the best mode of learning the sounds,—that is, of acquiring a sufficient number of sounds to serve as a point of departure, and guide for subsequent study. This method supposes that the sounds are analyzed and separated into their elements, and requires at first more than an ordinary share of attention; but the whole time required for learning the art is much shortened, and the subject greatly simplified.

Most of these initial sounds may be discovered in the healthy body,—that is, sounds sufficiently similar to those you meet with in disease to enable you to recognise them when they are met with; and if these are thoroughly learned, the remaining sounds, which are the most easy, are quickly acquired. You will find it to your advantage to follow very nearly the process which I shall point out, for the purpose of simplifying those sounds; for although it is less indispensably necessary, it is highly useful, and really will shorten the time and attention required.

In studying the physical signs, I follow as nearly as possible the natural method, reserving to myself, however, the privilege of deviating from it as often as may be advisable.

The physical signs, properly speaking, may be classed under the heads of alteration in the conformation of the thorax, resonance of the chest on percussion, and the sounds yielded by respiration, or produced during the act of coughing or speaking. These constitute the signs which may be regarded as strictly physical. There are some other signs, which, although less important, are, to a certain extent, classed among the physical signs; they belong more properly to the conformation of the thorax, than to any other division: under this head I shall treat of them. These are, percussion, or giving to the patient a sudden shake, to ascertain the presence of air and liquid in the cavity of the pleura, which is rarely practised, and is, in the majority of cases, both totally unnecessary and highly disagreeable to the patient; palpation, or examining the chest by placing the hands upon it, and pressing them carefully along the lateral portions of it.

### CONFORMATION OF THE THORAX.

The thorax, it is well known, resembles an irregular truncated cone. It is flattened on each side, and presents numerous inequalities, depressed in one part, and elevated in another. For the convenience of study, it is usual to examine it anteriorly, posteriorly, and laterally. Of these surfaces, the lateral, or the axillary, are the most regular,—the posterior, the least so. The walls of the thorax do not represent precisely the space occupied by the lungs and heart; for the liver, spleen, and stomach, encroach slightly upon the lower part of the cavity. This is particularly the case with the liver, which rises on the posterior part of the chest, nearly half an inch higher than the corresponding boundary on the left side. On the whole, the lower boundary may be represented by a line drawn from the spinous process of the twelfth dorsal vertebra, to the lower bone of the sternum: on the left side the boundary begins also at the twelfth dorsal vertebra, but passes at a distance of half, or at least one-third of an inch higher, until it reaches the præcordial region. The lower boundary of the chest, as thus defined, is not always the same, as the size of the liver is of course variable, and the dimensions of the thorax are necessarily influenced by this circumstance. This line is not followed with perfect regularity, especially on the left side, where the heart passes a little beyond the limit of the adjoining part of the chest. At the upper boundary, the difference of the two sides is less; on the right it sometimes rises a little higher than upon the left, from the greater development of the muscles and bony parietes of the thorax on that side; but this difference is, in general, so slightly marked, as scarcely to attract attention. The lungs extend a little beyond the clavicles, especially during the act of full inspiration, not exceeding half an inch. At the posterior part of the chest: the upper boundary is formed by a line drawn from the upper dorsal vertebra, outwards and downwards towards the point of the shoulder.

When the conformation of the thorax is perfectly normal, it presents an irregular plane in each of its four ribs; but the angles of these planes are sufficiently rounded to retain a general conoidal shape. Each side of it offers several elevations and depressions; at the anterior part these correspond with peculiarities of form of the viscera, and are really formed by the parietes of the chest; these irregularities of form at the posterior part are owing, in great part, to the muscles, and to the scapulæ. The clavicles form a ridge, which is slightly arched; the space above them is therefore depressed, except the patient be extremely corpulent, or labour under a disease of the lung or pleura. Beneath the clavicle, another depression, but one much shallower, exists; it extends to the lower part of the second rib. The space below this depression is slightly and regularly convex as far as the upper edge of the liver; at that level there is, in many persons, on the right side, a slightly depressed line, which corresponds with the interval between the liver and the lungs, on the left side, in young persons. There is often a prominence corresponding to the heart; this is slightly marked, and never divided, as it is in cases of real disease of this organ, or effusions within its investing membrane.

The lateral portions of the chest are regularly bulging from the apex to the base; and as the walls are here thinner than elsewhere, and nearly without muscles, the external form corresponds nearly to the lungs.

The posterior surface is rendered irregular by the scapulæ; but at the part uncovered by this bone its form is nearly as regular as that of the other portions, gradually widening towards the base of the chest. A slight depression, or gutter, exists on each side of the spine, for the reception of the dorsal muscles. The lower and posterior portions are often dilated from effusion into the pleura, and yield to the pressure of liquid with great readiness. The upper part is not changed in conformation, except the quantity of liquid be very large. The contraction of the chest is also extremely obvious at the lower portion after the absorption of pleuritic effusions.

In children the form of the chest is much more rounded than in adults; and in women, although the exterior seems more irregular than in males, yet the proper bony parietes are much more regularly formed, and are more conoidal in form.

The conformation of the chest, it is well known, is often characterized by individual peculiarities. Thus, some individuals are called chicken-breasted, from the prominence of the sternum, and others present a well-marked depression at the lower portion of this bone, which is sometimes congenital, and at other times is caused by trades or occupations which oblige the followers of them to work in a constrained posture, leaning forwards; this is particularly the case with shoemakers, who nearly all present this depression after working at their trade for a few years. Other individuals, who are thin, and of a feeble constitution, offer a remarkable contraction of the parietes of the chest; but, in all these cases, the contraction is more or less general, instead of being confined to a single part of the chest.—When it depends upon disease it is much more local, and is caused in nearly every instance by pleuritic adhesions, which draw the walls of the chest towards the lungs. Enlargement of the chest, beyond the natural average, is nearly as frequent as contraction. When it coincides with a general development of the body, and evidently depends upon a stout and large frame, it is of course indicative of health, rather than disease. The morbid dilatations, properly so called, are local, either limited to a part, or to the whole of one side of the chest; on this account they are readily recognised. They depend either upon an anormal development of these organs, or upon dilatations caused by effusions of air or liquid into the serous or mucous cavities of the chest. The comparison of the two sides is requisite, in order to recognise dilatations or contractions of the chest; and the thorax must be examined throughout in nearly every position, so that its true and relative dimensions may be ascertained.

It is not necessary that the chest should be exposed in order to examine its conformation, although this is much more convenient than to inspect it when covered. When no objections exist to exposing the chest, the patient should be placed in a sitting posture, or remain erect; if that be impossible, he should lie upon his back, and quite straight, so that the light may fall upon his chest: a cross light may of course give rise to error. The patient should then remain at rest, with his arms lying quietly by his sides, or slightly crossed, if the posterior part of his chest be examined; in this way the whole of the anterior or posterior surface may be taken in at a glance. An examination of this kind is, of course, not practicable, in cases of women, or of patients who are sweating profusely: under such circumstances, you must content yourself with the partial inspection which is practicable when the body is more or less covered by clothing, and you may aid in this examination by passing the hands lightly over the thorax. The examination by the touch is especially convenient for the posterior and lateral parts of the chest, where the morbid dilatation is generally most considerable.

The examination by the touch is called palpation, but I do not think it at all necessary to multiply terms in the description of the methods of physical examination. Palpation, then, is nothing more than the examination of the chest by means of the touch; it aids the sight, and often may be substituted for it when the patient is too thickly covered. The hand forms, as it were, a kind of natural callipers, and will give very accurate results. If you examine the lateral and inferior portions of the chest, you may place the whole palmar surface of the hand upon it; if the anterior and upper portions be examined, the fingers may be passed lightly over it. In this way you can detect any abrupt deviations from the natural conformation, but a general and moderate rise or depression cannot be detected except by the sight. If you cannot resort to this means of investigation, you must content yourselves with the other physical signs.

Dilatation of the chest is necessarily produced by all diseases which give rise to enlargement of the pulmonary vesicles, or to distention of the pleuræ. Those which act upon the pleuræ are inflammation, the products of which are serum, lymph, and purulent matter, or dropsy, in which the secreted fluid consists merely of serum. The effusions arising from pleurisy are nearly always confined to one side of the chest, take place rapidly, and are much more local than those of hydrothorax, which extend over a large surface, and are not confined to a single lung. Hence the pleuritic distention begins chiefly at the base of the lung, and extends upwards, involving the whole of one side only in these cases, in which the quantity is extremely great. Pericarditis gives rise to dilatation from the same cause as pleurisy, and the prominence follows very nearly the shape of the pericardium, and is therefore somewhat triangular, the small extremity upwards. The extreme dilatation which takes place in severe cases of pleurisy, in which the whole side of the chest is enlarged, elevates the shoulder, and gives the whole body an inclination towards the healthy side. This is often evident when the patient walks or sits in the erect posture. The effusions of liquid in the serous membranes give rise to the most decided, and, as it were, abrupt prominence of the chest; which the dilatation produced by enlarged vesicles is, in general, less decided, or, at least, more gradual. It gives rise to a more equable and moderate bulging of the chest, than that from effusions of liquid into the serous cavities. Of course it is most marked near those parts of the lung where the vesicles are most frequently dilated,—that is, along the anterior portion of the chest, on each side of the sternum; but, if it involve a large portion of the lungs, the shoulders are sometimes elevated, and the space above the clavicles becomes prominent, instead of offering a slight depression, as it does in the natural state.

Contraction of the thorax is a consequence of many diseases in which pleurisy has occurred, whether as a primary or secondary lesion; hence it is most marked in cases of primary pleurisy, especially where the quantity of effused liquid has been large. In the secondary pleurisy which follows or accompanies phthisis, contraction almost invariably takes place, and usually occurs near the summit of the lungs, so that the natural depressions, both above and below the clavicle, are exaggerated. Sometimes the depression reaches to the lower portions of the lung, as in ordinary pleurisy. The latter variety usually follows those cases of phthisical pleurisy which have commenced in the ordinary way, and in which the development of tubercles takes place rather late in the disease, after the inflammation has ceased, or at least has diminished. The general rule holds good, that contraction is evidence of previous pleurisy,—the exceptions are nearly all of a doubtful nature. In a few rare cases the tissue of the lung contracts from the partial or complete cicatrization of a cavity, or of an inflammation, although the attendant pleurisy may not be sufficiently extensive, or the adhesions strong enough to account for the depression. In these cases we are bound to admit that the pressure of the atmosphere has filled up the vacuum which would otherwise have been left. In the depression which follows pleurisy, it is true that the process is somewhat similar, as I shall show when speaking of this disease, but it is less strictly physical, and more dependent upon the contractile power of the adhesions. The absorption of the effused liquid in pericarditis does not give rise to a decided depression; it sometimes exists, but only in a slight degree.

I have given to you the general indications derived from an examination of the form of the thorax, and rely, as you may observe, chiefly upon the results which are derivable from the sight and touch. In a few cases the chest may be measured on the two sides, in order to estimate the difference in the semi-circumference more exactly, by passing a tape around the thorax, from the extremity of the spinous process of a vertebra, then marking the point corresponding to the middle of the sternum, and afterwards comparing the two parts extending from the sternum to the spine together. The seventh or eighth dorsal vertebra is the most convenient for this purpose. The measurement which is thus obtained is, of course, correct; but it applies only to those cases in which the difference is very evident, unless the dilatation occurs in the left. In the latter case the increased dimensions are readily perceived; for the right side is naturally larger than the left, and the difference is more or less according to the habits which the individual may have of exercising the right arm, more than the left: a difference in favour of this side would therefore be comparatively of little moment. Mensuration is of little value.

There is another mode of exploration which is termed succussion; it belongs to this part of the course as properly as to any other. I use the term merely to explain to you the method of performing it, not to advise you to resort to it. The method itself is sufficiently simple, and consists merely in placing the hands on the shoulders of the patient, and giving him a sudden jerking motion. If both air and liquid are contained in the cavity of the pleura, a gurgling, almost a splashing sound, is produced. There are other methods of investigation which are sufficient to make this state of things perfectly evident, so that we need not in any case resort to succussion.

